# Fermented Navy Bean-Derived Agents Restore Antibiotic Susceptibility in *Escherichia coli* Persisting on Fresh Produce after Washing through Transcriptional Modulation of Genes Associated with an *rpoS*-Centered Regulatory Network

**DOI:** 10.4014/jmb.2607.07019

**Published:** 2026-08-12

**Authors:** Sojeong Park, Se-Young Cho, Chyer Kim, Duwoon Kim

**Affiliations:** 1 Department of Integrative Food, Bioscience and Biotechnology, College of Agriculture and Life Sciences, Chonnam National University, Gwangju 61186, Republic of Korea; 2 Foodborne Virus Research Center, Chonnam National University, Gwangju 61186, Republic of Korea; 3 Agriculture Research Station, Virginia State University, Petersburg, VA 23806, USA; 4 Department of Food Science and Technology, College of Agriculture and Life Sciences, Chonnam National University, Gwangju 61186, Republic of Korea

**Keywords:** Washing-induced stress, *rpoS* stress-response network, PCC13-62-derived peptide, Bacterial adhesion regulation, Multidrug efflux pumps, Ampicillin resistance

## Abstract

Mechanical washing is an essential practice for reducing microbial contamination on fresh produce, yet the physiological adaptation of bacteria that persist after washing remains poorly understood. Here, we investigated the adaptive responses of lettuce-associated *Escherichia coli* ATCC25922 that remained attached after mechanical washing and evaluated the effects of fermented navy bean extract (FBE) and a PCC13-62-derived synthetic peptide (MFP-1). Saline washing detached only 25.1±3.9% of the attached bacterial population as the wash-removed population (WRP), while the majority of cells remained as a surface-retained population (SRP). Compared with the WRP, the SRP exhibited markedly higher survival following ampicillin treatment, accompanied by coordinated upregulation of stress-response genes (*rpoS*, *dnaK*, and *dps*), adhesion (*csgD*), and multidrug efflux (*marA*, *acrA*, *acrB*, and *tolC*) genes. In contrast, washing with FBE or MFP-1 at 200 μg/mL significantly increased recovery of the WRP to 80.8±5.4% without affecting bacterial viability, while suppressing expression of these stress-response, adhesion, and multidrug efflux genes in the ampicillin-resistant SRP (AMP^R^-SRP), restoring ampicillin susceptibility, and thereby reducing AMP^R^-SRP survival following ampicillin treatment to 2.4–2.8 log CFU/mL. These findings indicate that bacteria remaining attached after washing is associated with enhanced antibiotic resistance and concerted transcriptional activation of a proposed *rpoS*-centered regulatory network. FBE and MFP-1 effectively restored antibiotic susceptibility associated with transcriptional suppression of genes involved in this adaptive response, highlighting their potential as functional washing additives for improving the microbial safety of fresh produce.

## Introduction

The persistence of bacterial pathogens on fresh produce remains a major challenge for food safety, as plant surfaces provide heterogeneous microenvironments that facilitate microbial attachment and survival throughout the food supply chain [1-5]. Among produce-associated pathogens, pathogenic *Escherichia coli* (*e.g.*, *E. coli* O157:H7) is one of the major causes of foodborne illness [6]. Owing to the extensive genetic and physiological knowledge available for *E. coli*, this organism is also widely used as a model to investigate bacterial adaptation and persistence on fresh produce. Despite advances in decontamination technologies, bacterial adaptation to environmental stress continues to limit effective control [7]. For most consumers, washing is the primary decontamination practice performed before the consumption of fresh fruits and vegetables and serves as the first line of defense against microbial contamination. Although this process effectively removes a substantial proportion of microorganisms, surface-associated bacterial cells that remain attached are inevitably exposed to mechanical and physicochemical stresses, including shear force, osmotic changes, and oxidative conditions, which can alter bacterial physiology [8-11]. Importantly, exposure to such sublethal environmental stresses has been shown to trigger adaptive responses that enhance bacterial survival, surface persistence, and antimicrobial tolerance [8, 9]. Unlike planktonic cells, bacteria attached to produce surfaces require stable adhesion to persist on fresh produce despite repeated washing [12, 13]. Consequently, the detachment forces imposed during washing may represent an environmental challenge unique to surface-associated cells, extending beyond a simple physical removal process. Such attachment-challenging forces have the potential to elicit adaptive physiological responses analogous to those induced by other sublethal environmental stresses [8, 9]. In this study, the term washing-induced stress refers to the physiological response elicited by the attachment-challenging mechanical forces experienced by surface-associated bacterial cells during washing.

A central regulator of bacterial stress adaptation is the alternative sigma factor RpoS, which integrates environmental signals into transcriptional programs that promote survival under adverse conditions [14-16]. RpoS-mediated responses include activation of protective systems such as DnaK and Dps, which maintain protein homeostasis and genomic stability during environmental stress [17-20]. RpoS signaling is also linked to surface persistence through regulation of the transcription factor CsgD, the master regulator of curli production and biofilm formation [21, 22]. In parallel, stress-response pathways are closely associated with antibiotic resistance. The transcriptional regulator MarA activates multidrug efflux systems such as AcrAB–TolC, which reduce intracellular antibiotic accumulation [23-26]. Previous studies suggest that RpoS-mediated stress signaling interacts with multidrug resistance pathways, indicating that environmental stress may co-ordinately regulate bacterial persistence and antibiotic resistance through an integrated RpoS-centered regulatory network. However, whether washing-induced stress activates this regulatory hierarchy in produce-associated *E. coli* remains unclear [27, 28]. Although adaptive responses mediated by the RpoS-centered regulatory network under various environmental stresses have been extensively investigated [11, 27, 28], whether washing-induced stress experienced by surface-associated bacterial cells during washing activates this regulatory network remains unknown. We therefore hypothesize that bacteria remaining attached after washing exhibit concerted transcriptional responses associated with RpoS-dependent adaptation, thereby enhancing bacterial surface persistence and contributing to the development of antibiotic tolerance following washing.

Given that bacterial adaptation during washing may depend not only on the washing process itself but also on the properties of the washing agent, identifying washing agents capable of modulating bacterial stress responses represents a promising strategy for improving produce safety. In this regard, fermented navy bean extract (FBE), produced through *Bacillus* fermentation, is a complex mixture of fermentation-derived proteins, peptides, and metabolites. Our previous study demonstrated the antiviral activity of the FBE, suggesting that FBE-associated components may interact with viral surfaces [29]. Because FBE is a complex mixture of proteins and other bioactive constituents, these findings prompted us to explore whether its protein components could also provide peptide candidates capable of interacting with bacterial cells. Rather than focusing exclusively on the lectins and seed-associated proteins that predominated among the previously characterized FBE components, we investigated the desiccation-related protein PCC13-62 as a less-characterized source of functional peptides. PCC13-62 belongs to a protein family associated with plant responses to water deficit and osmotic stress [30], although its biological functions beyond these stress-related roles remain poorly understood. Candidate sequences derived from PCC13-62 were therefore explored as potential bioactive peptides, leading to the selection of MFP-1 for evaluation of its effects on bacterial detachment and antibiotic susceptibility. In particular, short bioactive peptides have attracted increasing attention as selective modulators of intracellular protein-protein interactions and regulatory pathways [31, 32], providing a promising strategy for controlling bacterial physiology without directly compromising cell viability. Accordingly, a peptide derived from PCC13-62, designated MFP-1, was evaluated together with FBE as a potential functional washing additives capable of promoting bacterial removal and mitigating washing-associated stress adaptation.

Accordingly, this study investigated whether bacterial populations retained on fresh produce after washing exhibit concerted transcriptional changes in genes associated with an *rpoS*-mediated regulatory network linking stress adaptation, adhesion, and ampicillin resistance in *E. coli*. We also evaluated the potential of FBE and MFP-1 as washing agents to enhance bacterial detachment, transcriptionally modulate genes associated with the proposed *rpoS*-mediated regulatory network and restore susceptibility to ampicillin.

## Materials and Methods

### Bacterial Strain and Culture Conditions

*Escherichia coli* ATCC 25922 (*E. coli*) was used as the model bacterial strain. The strain was cultured in tryptic soy broth (TSB) or Luria-Bertani (LB) broth at 37°C with shaking at 120 rpm. Overnight cultures were harvested by centrifugation at 5,000 × *g* for 10 min, washed twice with sterile 0.85% NaCl buffer, and the bacterial suspension was adjusted to an optical density at 600 nm (OD_600_) of 0.35, corresponding to 2 × 10^8^ CFU/mL. TSB, tryptic soy agar (TSA), LB, LB agar, and eosin methylene blue (EMB) agar used for bacterial culture and selective cultivation were purchased from Difco (USA). All other reagents were of analytical grade, and sterile distilled water was used throughout the experiments.

### Washing-Induced Stress Model and Selection of Stress-Adapted *E. coli* Populations

A washing-induced stress model was established to generate phenotypically distinct *E. coli* populations associated with lettuce. Fresh lettuce (*Lactuca sativa*) was purchased from a local market and used within 2 days. Inner leaves without visible damage were aseptically cut into 4 × 4 cm pieces, washed with sterile distilled water at room temperature (23 ± 2°C), and exposed to ultraviolet (UV) light for 40 min for surface sterilization. All steps were performed under an aseptic technique in a biological safety cabinet. An overnight culture of *E. coli* was prepared as described above and diluted to a final concentration of 2 × 10^6^ CFU/mL. Cut lettuce samples (2 g) were immersed in the diluted bacterial suspension and shaken at 100 rpm for 10 min to allow bacterial attachment. After inoculation, the samples were air-dried under biological safety cabinet for 1 h to stabilize bacterial association with the lettuce surface. This population was designated as the parental planktonic strain (PP) and served as the baseline inoculum. To induce washing-related environmental stress, inoculated lettuce samples were transferred into sterile bags containing 28 mL of 0.85% NaCl buffer and shaken at 120 rpm for 5 min. Bacteria released into the washing solution were collected and designated as the wash-removed population (WRP). Subsequently, washed lettuce samples were transferred into sterile stomacher bags containing 58 mL of 0.85% NaCl buffer, and bacteria remaining associated with the lettuce surface were recovered by homogenization using a stomacher device (LS-700A, InterScience, Saint-Nom-la-Bre) for 4 min. These bacteria were designated as the surface-retained population (SRP). To select bacterial populations resistant to ampicillin exposure following washing-induced stress, the SRP was inoculated into LB broth supplemented with ampicillin (8 μg/mL; Sigma-Aldrich, USA) and incubated at 37°C for 24 h with shaking at 120 rpm. SRP cells that survived under these conditions were designated as the ampicillin-resistant surface-retained population (AMP^R^-SRP). These stress-adapted populations were used for subsequent FBE and MFP-1 intervention experiments.

### Preparation and Processing of FBE

Navy bean (*Phaseolus vulgaris*) was purchased from a local market and used as the raw material for the preparation of FBE. The beans were thoroughly washed with distilled water to remove surface debris and subsequently surface-sterilized by immersion in a sodium hypochlorite solution (0.01%, v/v) for 5 min, followed by extensive rinsing with sterile distilled water. The cleaned beans were dried under hot-air conditions at 60°C for 72 h and then ground into a fine powder using a grinder (Youngjin Food Machinery, Republic of Korea). For fermentation, navy bean powder was added at a final concentration of 2% (w/v) to a minimal medium and inoculated with *Bacillus subtilis* strain. The culture was incubated under aerobic shaking conditions at 37°C and 150 rpm for 5 days. During fermentation, the dissolved oxygen level was maintained at 20–40% saturation, and the pH was adjusted to 7.0 using an Orion^TM^ Star A211 benchtop pH meter (Thermo Fisher Scientific, USA). After fermentation, the culture broth was centrifuged at 12,000 rpm for 15 min at 4°C to remove bacterial debris. The resulting supernatant was collected and filtered through a 0.22 μm polysulfone membrane filter (Millipore, USA). The filtered FBE was stored at -80°C until further use.

### Design and Synthesis of the PCC13-62–Derived Peptide MFP-1

A peptide was designed using the desiccation-related protein PCC13-62 (UniProt ID: V7BN55) as the source protein. Candidate peptide sequences were generated from the PCC13-62 amino acid sequence and evaluated based on sequence-derived physicochemical properties, including solubility, amino acid composition, and hydrophobicity, together with structural modeling using AlphaFold 2. Because Gram-negative bacterial surfaces are predominantly negatively charged, peptide candidates enriched in positively charged residues, particularly lysine (K) and arginine (R), were preferentially considered to promote electrostatic interactions with bacterial membranes. Based on these physicochemical and predicted structural characteristics, a representative peptide, designated MFP-1 (palmitoyl-KGGVPGGFYPKGGSGR), was selected for subsequent biological evaluation. N-terminal palmitoylation was introduced to enhance membrane affinity and peptide interaction with bacterial membranes. The peptide was synthesized by a commercial service (Dandi Cure Co., Ltd., Republic of Korea) using standard solid-phase peptide synthesis and purified to > 95% by HPLC.

### Determination of Concentration Ranges of FBE and MFP-1 without Growth Inhibition

To define concentration ranges suitable for subsequent treatment experiments, the effects of FBE and MFP-1 on basal bacterial growth were evaluated using the PP cells. This assessment was performed under non-stress conditions to establish FBE and MFP-1 concentrations appropriate for subsequent washing-induced stress or antibiotic exposure experiments. PP cells with 2 × 10^6^ CFU/mL were inoculated into LB medium containing FBE or MFP-1 at final concentration of 25, 50, 100, 200, 300, and 400 μg/mL. Cultures were incubated at 37°C with shaking at 120 rpm for 24 h. Bacterial growth kinetics were monitored by measuring OD_600_ at 4 h intervals using a microplate reader (Synergy, BioTek Instruments, USA). To quantify viable cell counts and corroborate OD_600_-based growth measurements, samples collected at the end of incubation were serially diluted and spread-plated on LB agar, followed by incubation at 37°C for 24 h, after which viable colonies were enumerated.

### Quantification of Bacterial Detachment Efficiency during Washing under FBE and MFP-1 Treatment

To quantify bacterial detachment rate during washing, PP cells attached to lettuce surfaces were subjected to washing in the presence or absence of FBE and MFP-1. Lettuce samples carrying attached PP cells were transferred into sterile bags containing 28 mL of washing solutions consisting of FBE or MFP-1 at final concentrations of 0, 25, 50, 100, and 200 μg/mL. Washing was performed by shaking at 120 rpm for 5 min. Following washing, detached and surface-retained bacterial fractions were recovered as described above. The washed solutions were serially diluted, spread-plated on TSA, and held at room temperature (23 ± 2°C) for 1h prior to overlaying with EMB agar, and incubated at 37°C for 24 h. Viable colonies were enumerated as colony-forming units (CFU), and these values were used to calculate detachment efficiency. Detachment efficiency (%) was calculated using the following equation:

$$\text{Detachment efficiency (\%)}=\frac{N_{washed}}{N_{washed}+N_{residual}}\times 100$$

where *N_washed_* represents the CFU of bacteria recovered from washing solution, and *N_residual_* represents the CFU of residual bacteria remaining on the lettuce surface after washing.

### Effects of FBE and MFP-1 on Ampicillin Susceptibility of Stress-Adapted AMP^R^-SRP

The AMP^R^-SRP generated by the washing-induced stress model was used for antibiotic exposure experiments in the presence or absence of FBE and MFP-1. AMP^R^-SRP cells were pre-cultured in LB medium and adjusted to an initial concentration 2 × 10^6^ CFU/mL. Cells were inoculated separately into LB medium supplemented with FBE or MFP-1 alone (25, 50, 100, or 200 μg/mL), AMP alone (8 μg/mL), FBE or MFP-1 under concurrent exposure to AMP. Cultures were incubated at 37°C for 24 h with shaking at 120 rpm. After incubation, samples were serially diluted with 0.85% NaCl buffer and spread-plated onto LB agar. Plates were incubated at 37°C for 24 h, after which colonies were enumerated as CFU/mL.

### Gene Expression Analysis of RpoS-Associated Regulatory Networks by qRT-PCR

*E. coli* populations subjected to quantitative real-time Polymerase Chain Reaction (qRT-PCR) analysis included the PP cells, WRP cells, SRP cells, and AMP^R^-SRP cells. In addition, AMP^R^-SRP cells recovered after co-treatment with AMP in combination with FBE or MFP-1 were also included for gene expression analysis. qRT-PCR was performed to evaluate the expression of genes associated with stress response, surface adhesion, and antibiotic resistance. Collected bacterial cells were centrifuged at 5,000 × g for 10 min at 4°C, after which the supernatant was discarded. The resulting cell pellets were washed twice with 0.85% NaCl buffer to remove residual medium components and subsequently used for RNA extraction. Total RNA was extracted using RNAiso Plus (Takara Bio, Japan) according to the manufacturer’s protocol, including chloroform extraction, isopropanol precipitation, 75% ethanol washing, and resuspension in RNase-free water. RNA concentrations were determined using a Nano Drop^TM^ One microvolume UV-Vis spectrophotometer (Thermo Scientific^TM^, USA). Reverse transcription was carried out with PrimeScript Reverse Transcriptase (Takara, Japan). Briefly, RNA was denatured with 6-mer random primers at 65°C for 10 min, then reverse transcribed in a mixture containing 5× PrimeScript RT Master Mix, 5× DTT, dNTPs, PrimeScript RTase, and RNase inhibitor at 37°C for 1 h, followed by enzyme inactivation at 95°C for 5 min. The resulting cDNA was used for qRT-PCR using gene-specific primers (Table 1). The qRT-PCR was performed on a Thermal Cycler Dice Real-Time System III (Takara Bio) under the following conditions: initial denaturation at 95°C for 10 min, followed by 45 cycles of denaturation at 95°C for 5 s, annealing at 58°C for 10 s and extension at 72°C for 20 s. Gene expression levels were analyzed using the 2^−ΔΔCt^ method, with 16S rRNA used as the internal reference gene. Relative expression levels were normalized to the untreated control group.

### Statistical Analysis

All experiments were performed in triplicate, and the results are presented as mean ± standard deviation (SD). Statistical analyses and graphical visualizations were performed using GraphPad Prism for Windows (version 8.1.1; GraphPad Software, Inc., USA). Prior to ANOVA, data were evaluated for normality using the Shapiro-Wilk test and homogeneity of variance using the Brown-Forsythe test. Statistical significance was determined using one-way analysis of variance (ANOVA) with Dunnett’s post hoc test for comparisons against controls, and two-way ANOVA with Bonferroni’s post hoc test for analyses involving two variables. Asterisks denote statistical significance (* *P* < 0.05, ** *P* < 0.01, *** *P* < 0.001; ns, not significant).

## Results

### Bacterial Detachment and Adjuvant Effects on Washing-Induced AMP Resistance

**Fig. 1:**
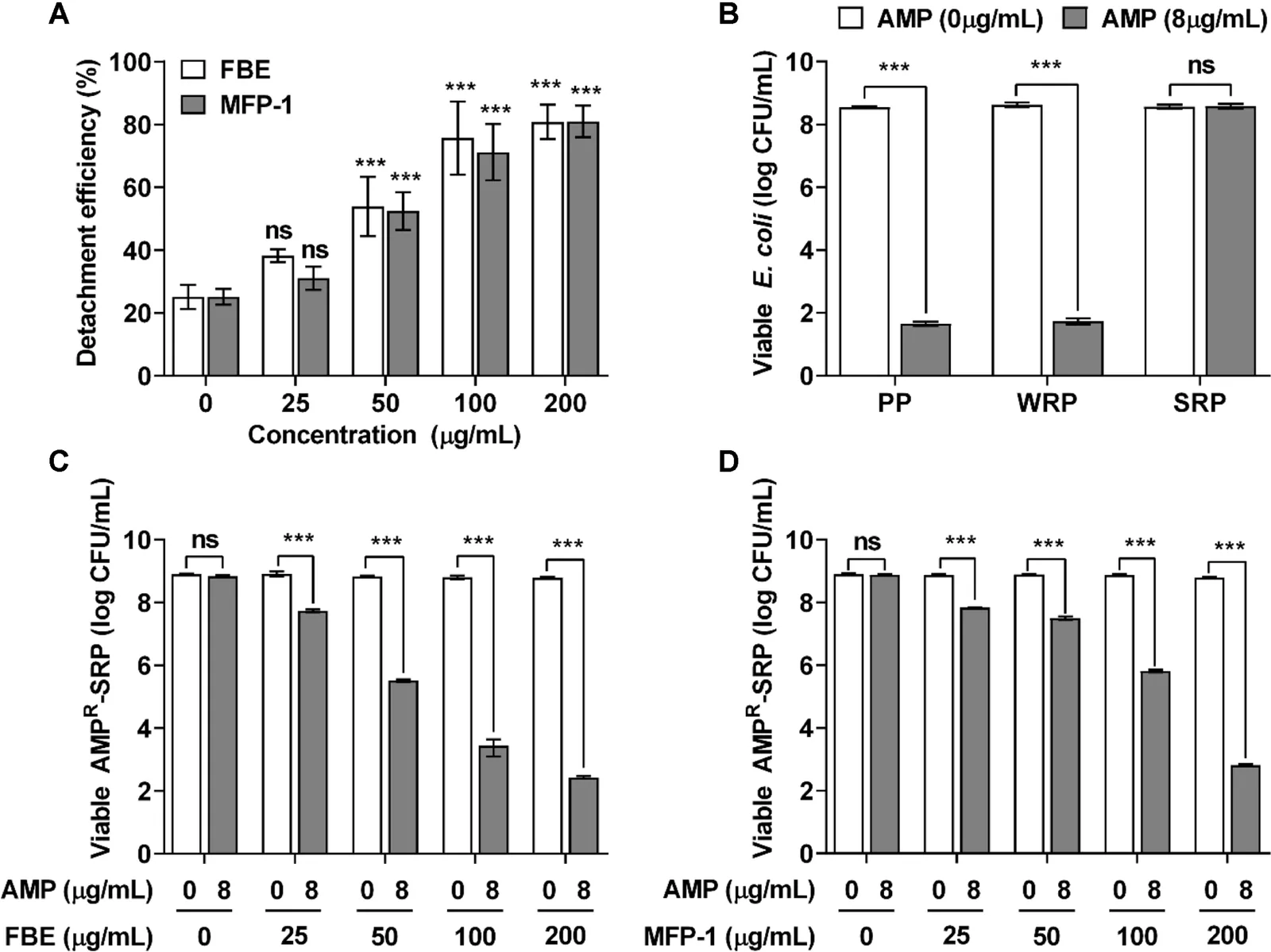
FBE and MFP-1 promote bacterial detachment and restore antibiotic susceptibility in stress-adapted *Escherichia coli*. (**A**) Detachment efficiency (%), calculated as percentage of the initially attached *E. coli* population recovered as the wash-removed population (WRP), following treatment with fermented navy bean extract (FBE) or PCC13-62-derived peptide (MFP-1) (25–200 μg/mL). (**B**) Survival of parental planktonic (PP), wash-removed population (WRP), and surface-retained population (SRP) following exposure to ampicillin (8 μg/mL). (**C, D**) Viable cell counts of ampicillin-resistant SRP (AMP^R^-SRP) following co-treatment with ampicillin (8 μg/mL) and FBE (0–200 μg/mL) or MFP-1 (0–200 μg/mL). Data were analyzed by two-way ANOVA followed by Bonferroni’s post hoc test. Asterisks denote statistical significance (*P < 0.05, **P < 0.01, ***P< 0.001; ns, not significant).

Mechanical washing separated surface-associated *E. coli* into distinct bacterial populations with different responses to ampicillin. We first investigated the WRP, representing bacterial cells detached from the lettuce surface during washing. A known inoculum of planktonic *E. coli* cells was first allowed to attach to the lettuce surface, after which the lettuce was washed with either 0.85% NaCl as a control, FBE or MFP-1. Washing with 0.85% NaCl recovered 25.1 ± 3.9% of the initially attached cells as WRP, which was used to determine the detachment efficiency. Likewise, FBE or MFP-1 increased the proportion of WRP recovery, resulting in a concentration-dependent increase in detachment efficiency. For FBE the proportion of WRP increased to 53.9 ± 9.4%, 75.7 ± 11.6%, and 80.8 ± 5.4% at 50, 100, and 200 μg/mL, respectively. A comparable concentration-dependent increase was observed with MFP-1, which increased the proportion of WRP to 52.4 ± 6.0%, 71.2 ± 8.9%, and 81.0 ± 5.0% at the corresponding concentrations (Fig. 1A and Table S1). To compare antibiotic susceptibility among the bacterial populations, PP, WRP, and SRP were individually exposed to ampicillin. Following ampicillin treatment, viable cell counts of PP and WRP were similarly reduced to 1.7 ± 0.1 log CFU/mL, whereas SRP cells remained highly viable to 8.6 ± 0.1 log CFU/mL, demonstrating substantially greater tolerance to ampicillin (Fig. 1B). The effect of FBE and MFP-1 was subsequently evaluated using AMP^R^-SRP, the ampicillin-resistant subpopulation isolated from SRP. In the presence of ampicillin, FBE reduced viable AMP^R^-SRP cells in a concentration-dependent manner from 8.8 ± 0.02 log CFU/mL in the ampicillin-only control to 5.5 ± 0.04, 3.4 ± 0.31, 2.4 ± 0.05 log CFU/mL at 50, 100, and 200 μg/mL, respectively (Fig. 1C). Similarly, MFP-1 reduced AMP^R^-SRP viability to 7.5 ± 0.05, 5.8 ± 0.04, and 2.8 ± 0.04 log CFU/mL at increasing concentrations (Fig. 1D). Neither FBE nor MFP-1 alone affected bacterial viability, with viable cell counts remaining at 9.1–9.2 log CFU/mL across all tested groups (Supplementary Fig. S1), indicating that the reduced viability of AMP^R^-SRP cells in cell resulted antibiotic susceptibility rather than direct bactericidal activity.

### Transcriptional Profiles of Stress-Response, Adhesion, and Antibiotic Resistance Genes in SRP

**Fig. 2:**
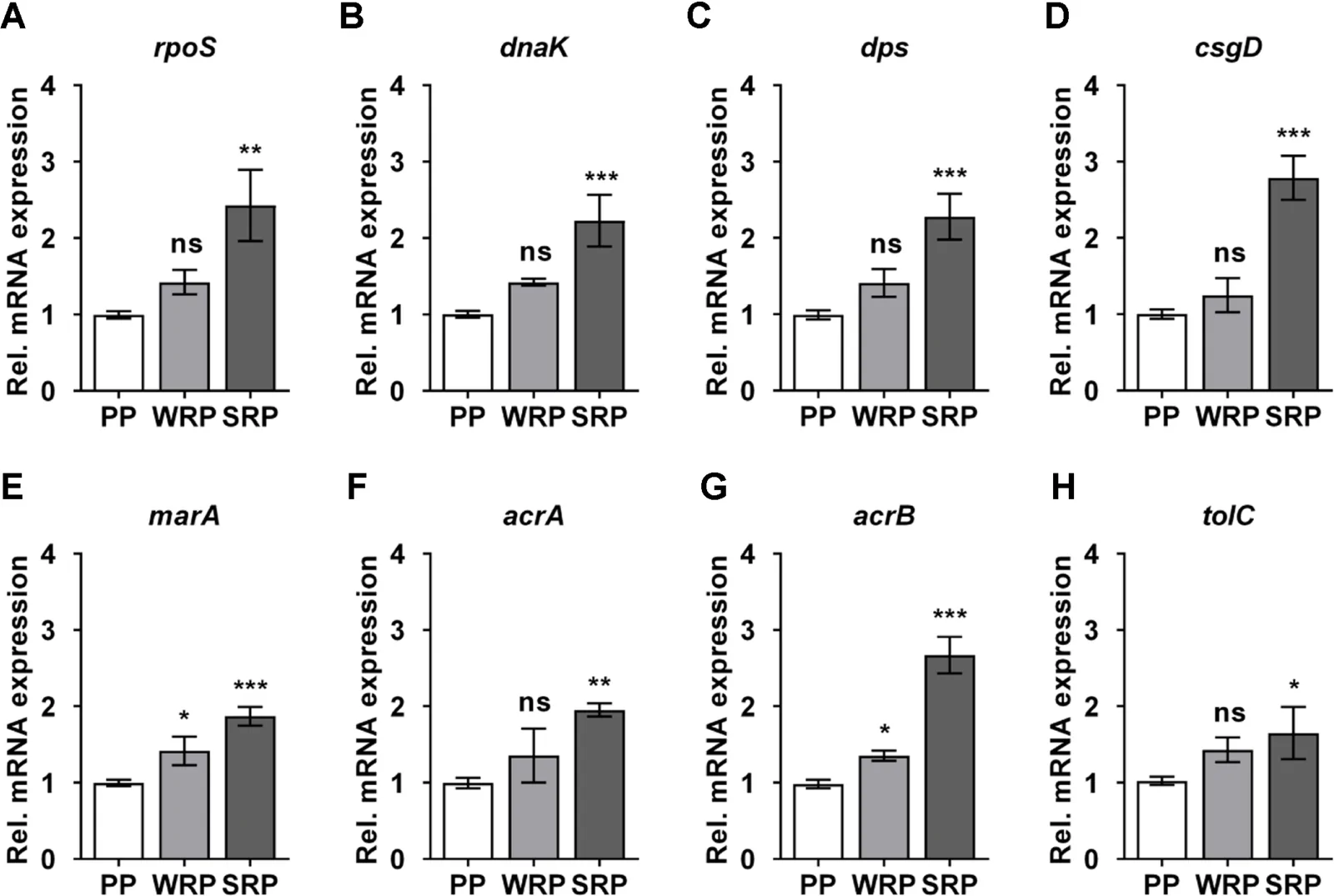
Transcriptional profiles of stress-response, adhesion, and antibiotic resistance genes in distinct *Escherichia coli* populations following washing stress. Relative mRNA expression levels of stress response genes (**A**) *rpoS* and (**B**) *dnaK*, oxidative stress (**C**) *dps*, adhesion regulator (**D**) *csgD*, multidrug resistance regulator (**E**) *marA*, and efflux pump components (**F**) *acrA*, (**G**) *acrB*, (**H**) *tolC* were quantified by qRT-PCR in parental planktonic (PP), wash-removed population (WRP), and surface-retained population (SRP). Gene expression levels were normalized to the reference gene 16S rRNA and expressed as fold change relative to PP cells. Data was analyzed by one-way ANOVA followed by Dunnett’s post hoc test, with comparisons made against the PP cells. Asterisks denote statistical significance (*P < 0.05, **P < 0.01, ***P < 0.001; ns, not significant).

Differences in ampicillin susceptibility among PP, WRP, and SRP prompted us to examine whether these populations also differed at the transcriptional level. Gene expression analysis focused on representative genes associated with stress response, adhesion, and antibiotic resistance. Among the three populations, SRP consistently exhibited the highest expression of stress response genes, including *rpoS* (2.4 ± 0.5-fold), *dnaK* (2.2 ± 0.3-fold), and *dps* (2.3 ± 0.3-fold), compared with PP (Fig. 2A–2C). Likewise, the adhesion regulator *csgD* was significantly upregulated in SRP cells (2.8 ± 0.3-fold) (Fig. 2D). Expression of antibiotic resistance–associated genes were also elevated in SRP, including *marA* (1.9 ± 0.1-fold) and efflux pump components *acrA* (2.0 ± 0.1-fold), *acrB* (2.7 ± 0.2-fold), and *tolC* (1.7 ± 0.3-fold) (Fig. 2E–2H).

### FBE and MFP-1 Suppress Stress Response and Adhesion Gene Expression in AMP^R^-SRP

**Fig. 3:**
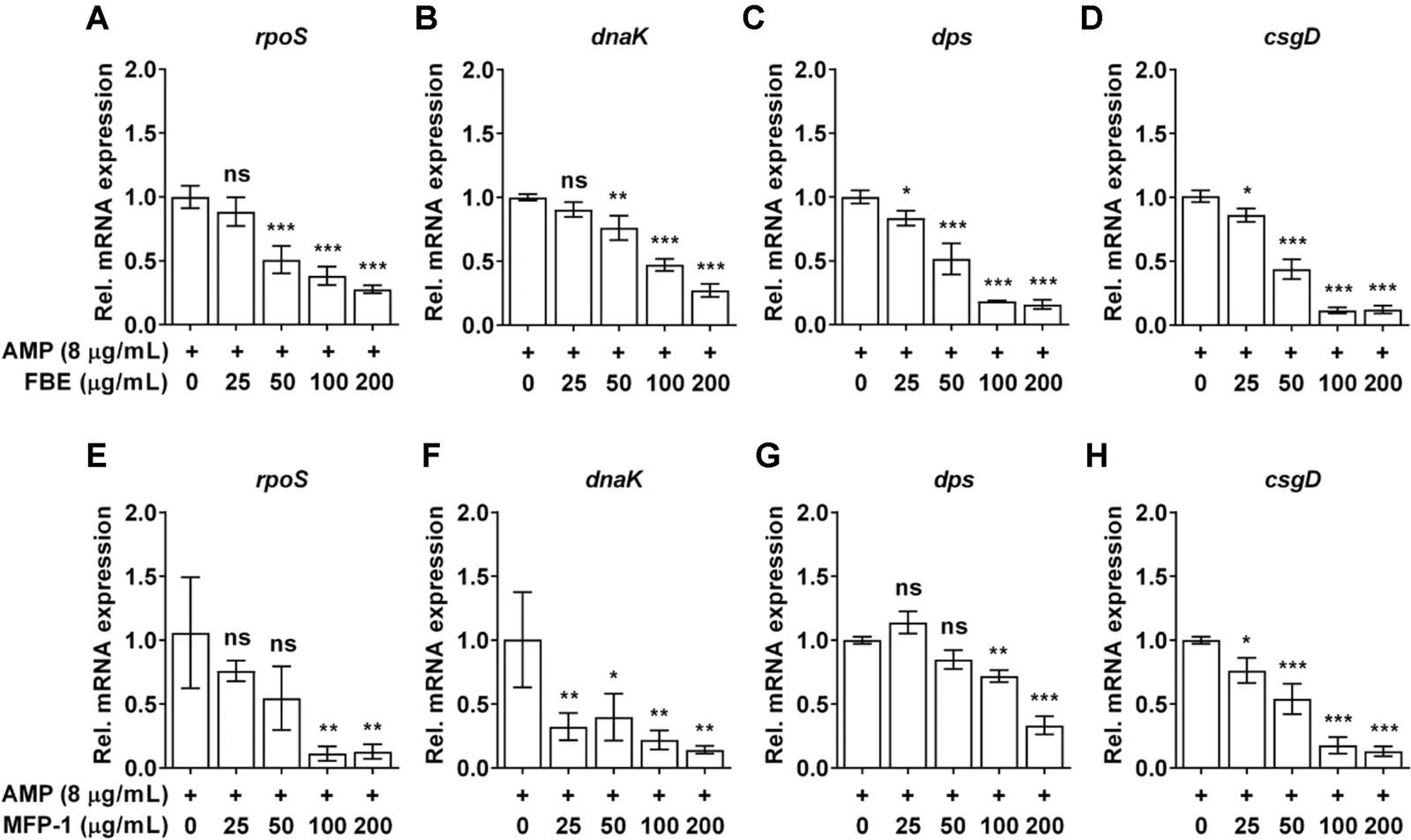
Transcriptional modulation of stress-response and adhesion–related genes in ampicillin-resistant surface-retained *E. coli* (AMP^R^-SRP) following FBE and MFP-1 treatment. Relative mRNA expression levels of *rpoS* (**A, E**), *dnaK* (**B, F**), *dps* (**C, G**), and *csgD* (**D, H**) were quantified by qRTPCR in AMP^R^-SRP cells treated with ampicillin (8 μg/mL) in the presence of fermented navy bean extract (FBE; 25−200 μg/mL) or PCC13-62-derived peptide (MFP-1; 25−200 μg/mL). Gene expression levels were normalized to the reference gene 16S rRNA and expressed as fold change relative to AMP-only control. Data was analyzed by one-way ANOVA followed by Dunnett’s post hoc test, with comparisons made against the AMP-only control. Asterisks denote statistical significance (*P < 0.05, **P < 0.01, ***P < 0.001; ns, not significant).

Given the coordinated upregulation of stress response and adhesion genes in SRP, we next investigated whether this stress-adapted transcriptional profile was maintained following ampicillin exposure and whether treatment with FBE or MFP-1 could reverse these transcriptional changes in the AMP^R^-SRP cells. FBE significantly suppressed the expression of the stress-response regulators *rpoS*, *dnaK*, and *dps*, as well as the adhesion regulator *csgD*, in a concentration-dependent manner (Fig. 3A–3D). At 200 μg/mL, expression of *rpoS*, *dnaK*, *dps*, and *csgD* decreased to 0.3 ± 0.03-, 0.3 ± 0.05-, 0.2 ± 0.04-, and 0.1 ± 0.03-fold, respectively, relative to untreated AMP^R^-SRP cells. MFP-1 produced a similar concentration-dependent inhibitory effect (Fig. 3E–3H). At 200 μg/mL, expression of *rpoS*, *dnaK*, *dps*, and *csgD* was reduced to 0.1 ± 0.06-, 0.1 ± 0.03-, 0.3 ± 0.07-, and 0.1 ± 0.04-fold, respectively, compared with untreated AMP^R^-SRP cells.

### FBE and MFP-1 Suppress Antibiotic Resistance Gene Expression in AMP^R^-SRP

**Fig. 4:**
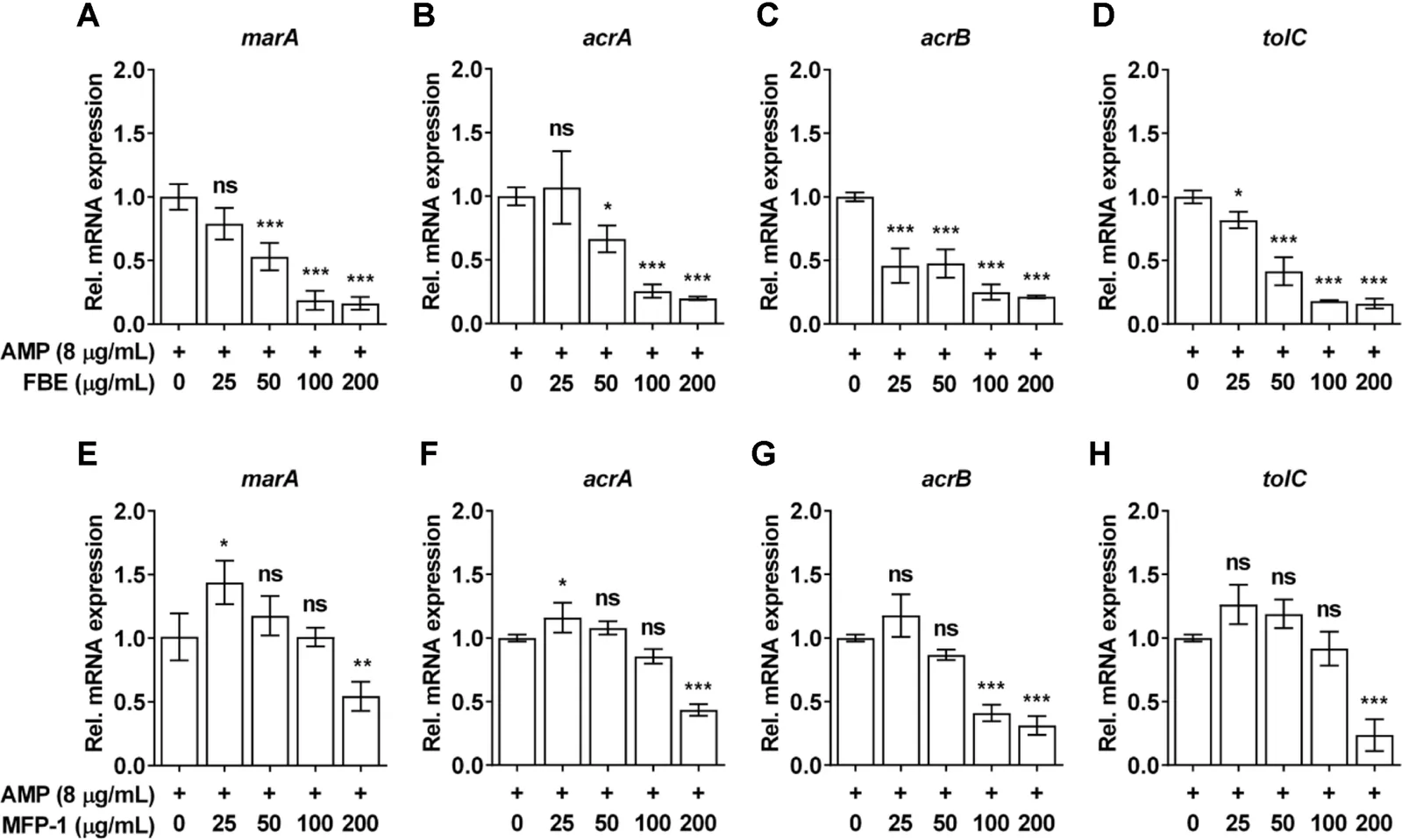
Transcriptional modulation of antibiotic resistance–related genes in ampicillin-resistant surface-retained *E. coli* (AMP^R^-SRP) following FBE and MFP-1 treatment. Relative mRNA expression levels of antibiotic resistance–associated genes such as *marA* (**A, E**), *acrA* (**B, F**), *acrB* (**C, G**), and *tolC* (**D, H**) were quantified by qRT-PCR in AMP^R^-SRP cells treated with ampicillin (8 μg/mL) in the presence of fermented navy bean extract (FBE; 25-200 μg/mL) or PCC13-62-derived peptide (MFP-1; 25-200 μg/mL). Gene expression levels were normalized to the reference gene 16S rRNA and expressed as fold change relative to AMP-only control. Data was analyzed by one-way ANOVA followed by Dunnett’s post hoc test, with comparisons made against the AMP-only control. Asterisks denote statistical significance (*P < 0.05, **P < 0.01, ***P < 0.001; ns, not significant).

Whether the transcriptional suppression induced by FBE and MFP-1 also extended to antibiotic resistance-associated genes was therefore investigated in AMP^R^-SRP cells. FBE treatment significantly suppressed the expression of *marA* and the AcrAB-TolC multidrug efflux pump genes (*acrA*, *acrB*, and *tolC*) in a concentration-dependent manner (Fig. 4A–4D). At 200 μg/mL, expression of *marA*, *acrA*, *acrB*, and *tolC* decreased to 0.2 ± 0.05-, 0.2 ± 0.01-, 0.2 ± 0.01-, and 0.2 ± 0.04-fold, respectively, relative to untreated AMP^R^-SRP cells. MFP-1 produced a similar concentration-dependent inhibitory effect (Fig. 4E–4H). At 200 μg/mL, expression of *marA*, *acrA*, *acrB*, and *tolC* was reduced to 0.5 ± 0.11-, 0.4 ± 0.04-, 0.3 ± 0.07-, and 0.2 ± 0.12-fold, respectively. Collectively, both FBE and MFP-1 consistently suppressed the expression of multidrug resistance-associated genes in AMP^R^-SRP.

### Transcriptional Changes associated with the Proposed *rpoS*-Centered Regulatory Network by FBE and MFP-1

**Fig. 5:**
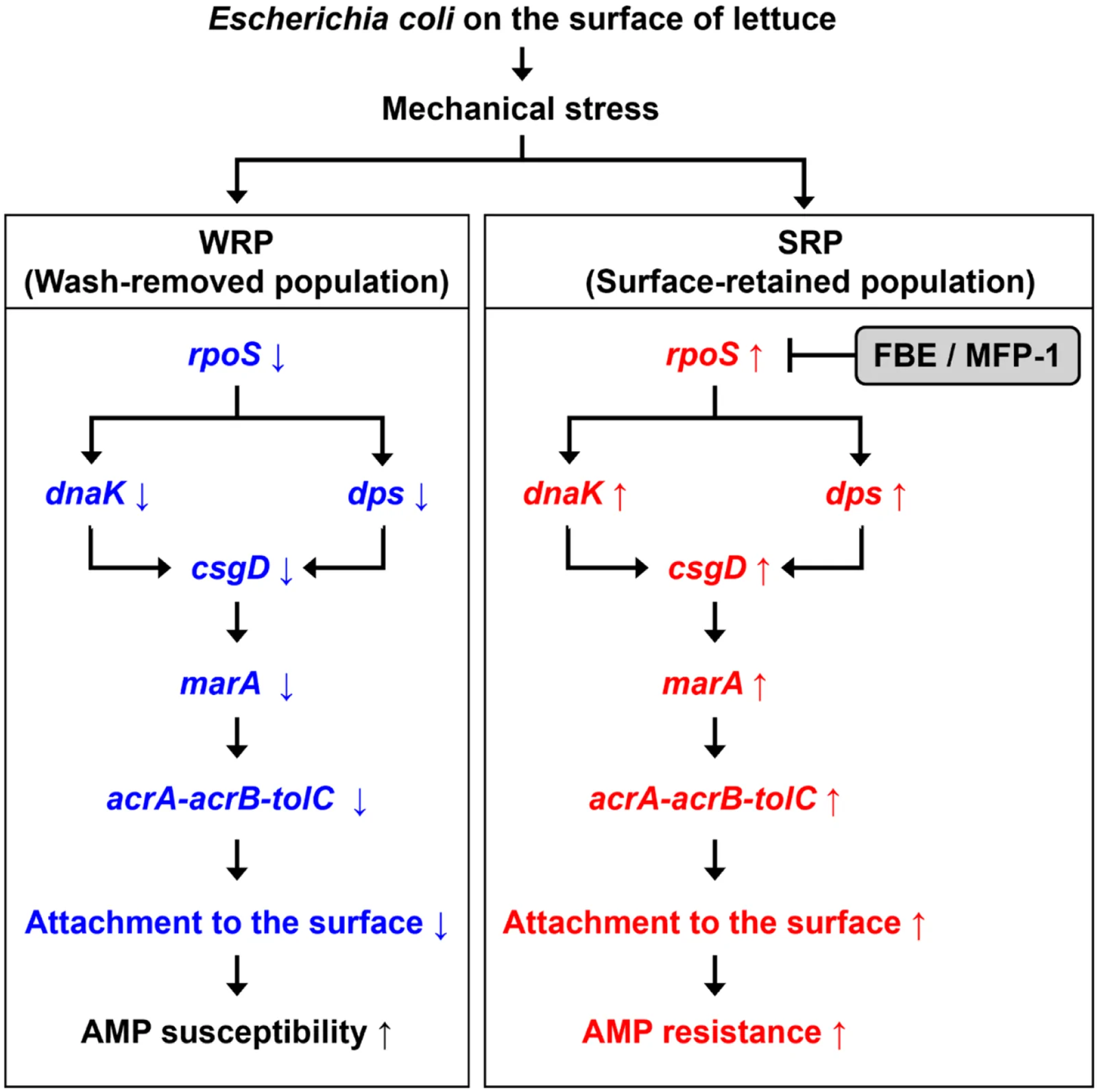
Proposed transcriptional model of *rpoS*-centered stress-adhesion-resistance network in surface-retained *E. coli* and its modulation by FBE and MFP-1. The schematic summarizes the concerted transcriptional responses observed in wash-removed and surface-retained *E. coli* populations following conventional washing. Based on the transcriptional changes observed in this study together with previously established regulatory relationships, increased expression of in the surface-retained cells exhibited increased expression of *rpoS*, accompanied by increased expression of the chaperone *dnaK*, and the oxidative stress protein *dps*. This stress-adapted state is associated with increased expression of the adhesion through upregulation of the curli and biofilm regulator *csgD*, while simultaneously together with increased expression of the multidrug resistance regulator *marA*, and the downstream efflux pump components *acrA*, *acrB*, and *tolC*. Together, this network establishes a surface-associated, stress-hardened phenotype characterized by enhanced persistence and antibiotic resistance.

Based on the transcriptional changes observed in this study, a proposed transcriptional model linking washing-induced stress, bacterial adhesion, and antibiotic resistance was constructed (Fig. 5). SRP exhibited coordinated upregulation of *rpoS* together with increased expression of the stress-response genes *dnaK* and *dps*, the adhesion regulator *csgD*, and the antibiotic resistance-associated genes *marA* and the *acrA*–*acrB*–*tolC* efflux operon. Among the regulatory genes examined, *rpoS* was positioned as the central node in the proposed model as its expression pattern paralleled those of the other stress-response, adhesion, and antibiotic resistance-associated genes following both mechanical washing and FBE/MFP-1 treatment. Treatment with FBE consistently suppressed the expression of these regulatory genes and was accompanied by enhanced bacterial detachment and restoration of ampicillin susceptibility. Notably, MFP-1, a peptide derived from FBE, produced transcriptional and phenotypic responses comparable to those of FBE, suggesting that MFP-1 may contribute to the transcriptional and phenotypic effects observed with FBE. Collectively, these findings indicate that FBE and MFP-1 are associated with transcriptional modulation of the proposed *rpoS*-centered regulatory network, accompanied by reduced bacterial adhesion and restoration of antibiotic susceptibility in the surface-retained bacterial population.

## Discussion

Conventional washing is generally regarded as a physical process that reduces bacterial contamination on fresh produce [33]. However, our findings demonstrate that the bacterial population remaining attached after conventional washing exhibited a distinct stress-adapted phenotype characterized by enhanced ampicillin resistance together with concurrent transcriptional changes in stress-response, adhesion, and multidrug resistance-associated genes. These observations suggest that bacteria remaining attached after washing represent a physiologically distinct bacterial population characterized by enhanced stress-adaptation and antibiotic resistance. In this context, FBE and MFP-1 promoted bacterial detachment and restored antibiotic susceptibility without affecting bacterial viability, suggesting that their effects are associated with modulation of stress adaptation rather than direct bactericidal activity. Consistent with this interpretation, both agents significantly increased WRP, resulting in higher detachment efficiency while maintaining bacterial viability. These findings suggest that these agents promote bacterial detachment associated with transcriptional changes in adhesion-related genes rather than through direct bactericidal activity. This mode of action is consistent with previous studies demonstrating that protein- and peptide-based anti-adhesion agents, including lectins, suppress bacterial adhesion and biofilm formation by targeting adhesion-associated processes without compromising bacterial viability [34, 35]. Notably, SRP cells that remained attached to the lettuce surface after washing exhibited markedly higher antibiotic resistance than WRP cells. Although both WRP and SRP originated from the same parental PP, which was initially susceptible to ampicillin, only the SRP exhibited enhanced antibiotic resistance following washing (Fig. 1B). These findings are consistent with the possibility that remaining attached after washing represents a stress-adapted subpopulation exhibiting enhanced antibiotic resistance. This phenotype is consistent with the previous reports demonstrating that exposure to environmental stress activates bacterial adaptative responses that enhance survival and promote cross-protection against subsequent environmental and antimicrobial conditions [14, 36]. Importantly, despite the increased antibiotic resistance of SRP cells, co-treatment with ampicillin and FBE or MFP-1 markedly reduced the viability of AMP^R^-SRP cells in a concentration-dependent manner. In contrast, treatment with ampicillin, FBE or MFP-1 alone had no significant effect on bacterial survival. These findings indicate that restoration of antibiotic susceptibility may result from modulation of regulatory pathways linking stress adaptation, bacterial adhesion, and biofilm-associated resistance mechanisms [37, 38].

To investigate the transcriptional basis associated with this phenotype, we examined the expression of key stress-response and adhesion-related, and antibiotic resistance-associated genes. The alternative sigma factor RpoS serves as a central regulator of bacterial general stress response, integrating environmental signals into transcriptional programs that promote survival under adverse conditions [14, 39]. Downstream of RpoS, stress-protective effectors such as the chaperone DnaK and the DNA-binding protein Dps contribute to proteostasis and genome protection under adverse conditions [18, 40]. In addition, CsgD has been reported to function as a master regulator of curli biosynthesis and extracellular matrix formation, thereby promoting bacterial surface attachment and persistence [21, 41, 42]. Consistent with these established roles, the downregulation of *dnaK*, *dps*, and *csgD* following FBE and MFP-1 treatment is consistent with reduced stress adaptation, and surface persistence, which may contribute to decreased biofilm-mediated antimicrobial tolerance [43, 44]. In parallel, multidrug resistance in *E. coli* has been reported to be mediated by the MarA-regulated AcrAB–TolC efflux system, which functions as a major multidrug efflux mechanism by limiting intracellular antibiotic accumulation [23, 24, 45, 46]. Functional cross-talk between bacterial stress-response pathways and multidrug resistance networks has also been reported [47, 48]. Accordingly, the downregulation of *marA*, *acrA*, *acrB*, and *tolC* following FBE and MFP-1 treatment is consistent with a transcriptional profile suggestive of reduced efflux activity, which may contribute to enhanced intracellular antibiotic retention and restored susceptibility. Consistent with this regulatory framework, SRP cells exhibited coordinated upregulation of *rpoS*, *dnaK*/*dps*, *csgD*, and *marA*–*acrA*B–*tolC* relative to WRP cells, suggesting that mechanical washing selected for a bacterial subpopulation in which stress adaptation and multidrug resistance are transcriptionally coupled.

Importantly, FBE and MFP-1 reversed this coordinated transcriptional program. Both agents suppressed the expression of *rpoS* and its downstream effectors *dnaK*/*dps* and *csgD*, consistent with enhanced bacterial detachment and reduced surface persistence. Although FBE and MFP-1 produced broadly similar regulatory effects, their effects on efflux-associated genes differed. FBE induced relatively uniform suppression across all target genes, whereas MFP-1 exhibited a more pronounced concentration-dependent response, particularly for efflux-associated genes. This transcriptional reprogramming corresponds well with both the reduced persistence of antibiotic-resistant surface-retained cells and the restoration of antibiotic susceptibility following FBE or MFP-1 treatment, supporting a model in which interference with the RpoS-centered stress–adhesion–resistance network reverses the stress-adapted phenotype of surface-retained *E. coli*. The present findings should be interpreted in the context of the cut lettuce model used in this study. Previous studies have shown that wounded leaf tissues can facilitate bacterial attachment and persistence compared with intact leaf surfaces [4, 49]. Because bacterial attachment may differ between wounded and intact leaf tissues, the absolute level of bacterial attachment observed in this study may not fully represent that intact produce surfaces. Nevertheless, all experimental groups, including the untreated control, were evaluated under identical conditions, allowing valid comparisons of the relative effects of FBE and MFP-1 on bacterial detachment and washing-induced adaptative responses. In addition, the proposed *rpoS*-centered regulatory network was inferred from concerted transcriptional responses, providing a framework for future studies incorporating protein-level analyses and functional validation.

Taken together, these findings suggest that although conventional washing is widely applied to reduce microbial contamination on fresh produce [50], attention should also be given to the bacterial population remaining attached after washing. In the present study, the surface-retained bacterial population remaining after conventional washing exhibited concerted transcriptional changes in stress response, adhesion, and multidrug resistance-associated genes together with enhanced ampicillin tolerance. These observations are consistent with previous evidence that environmental stress encountered during minimal processing can induce adaptive stress response in bacteria [51]. Our transcriptional data further supports a proposed regulatory network linking stress response, adhesion, and multidrug resistance. FBE and MFP-1 were associated with reversal of this coordinated transcriptional program at non-lethal concentrations, restoring antibiotic susceptibility without imposing direct selective pressure on bacterial viability. This finding suggests that targeting regulatory networks governing stress adaptation, bacterial adhesion, and antibiotic resistance, rather than bacterial viability itself, may represent a promising alternative antimicrobial strategy capable of enhancing antibiotic susceptibility while minimizing the selective pressures that drive resistance development [16, 48, 52-54].

## Conclusion

The surface-retained bacterial population of lettuce-associated *E. coli* following conventional washing exhibited enhanced ampicillin resistance together with concerted transcriptional changes in stress-response, adhesion, and multidrug resistance-associated genes. FBE and MFP-1 promoted bacterial detachment and restored ampicillin susceptibility without exerting direct bactericidal effects, accompanied by reduced expression of representative genes within the proposed *rpoS*-centered regulatory network. These findings provide transcriptional evidence supporting the involvement of the proposed network in the stress response, adhesion, and ampicillin-resistance characteristics of surface-retained *E. coli* and suggest the potential of FBE and MFP-1 as functional washing agents. The results further emphasize the importance of considering not only bacterial removal efficiency but also the characteristics of bacteria remaining attached after washing when evaluating produce washing strategies.

## Supplemental Materials

Supplementary data for this paper are available on-line only at http://jmb.or.kr.



## Figures and Tables

**Table 1:** Gene-specific primers used for qRT-PCR analysis in *E. coli*.

Gene	Primer	Primer sequence (5’ - 3’)
*16S rRNA*	Forward	TACCGCATAACGTCGCAAGA
	Reverse	TTCCAGTGTGGCTGGTCATC
*rpoS*	Forward	CAGGTGAGACAGGATCAGCG
	Reverse	CCTACATCCGGGCAGCTAAC
*dnaK*	Forward	TACGGTCTGGACAAAGGCAC
	Reverse	GGTGGGTATCACCGTTGGTT
*dps*	Forward	GTCGTCATCTTTCGCTTCGC
	Reverse	CAGCAAAACCCCGCTGAAAA
*csgD*	Forward	CGTAAAGTAGCATTCGCCGC
	Reverse	GATTACCCGTACCGCGACAT
*marA*	Forward	TCCAGACGCAATACTGACGC
	Reverse	GTTGCAGGTGCCATTTGGAG
*acrA*	Forward	TCTGGCAAGAAGGAGCTTGG
	Reverse	AGAAGAGCACGCACCACTAC
*acrB*	Forward	AGGAAGTCGTTGCTGGACTG
	Reverse	CACCTCTCCGATTAGCGGTC
*tolC*	Forward	CAGGATCTGCTGGCACTGAA
	Reverse	GCGGATGTTTGCTGAACGAC
