# Supplementary material for: Fermented Navy Bean-Derived Agents Restore Antibiotic Susceptibility in Escherichia coli Persisting on Fresh Produce after Washing through Transcriptional Modulation of Genes Associated with an rpoS-Centered Regulatory Network
Source: J Microbiol Biotechnol. 2026 Aug 12;36:e2607019. doi: 10.4014/jmb.2607.07019 (PMC13500918; doi:10.4014/jmb.2607.07019)
Supplement: Supplementary file 1 [file jmb-36-e2607019-supple.pdf]

J. Microbiol. Biotechnol. 2026. 36: e2607019
https://doi.org/10.4014/jmb.2607.07019

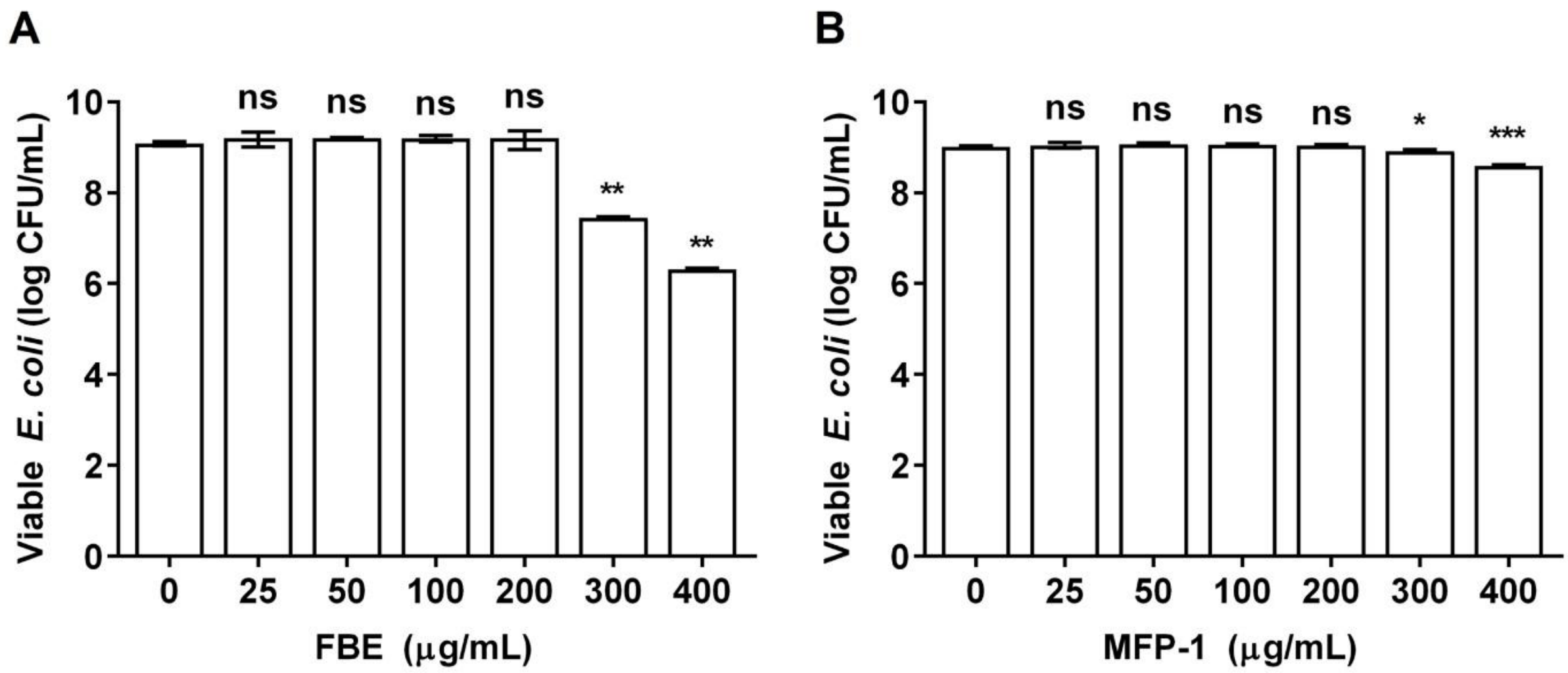

Fig. S1. Effects of Fermented navy bean extract (FBE) and PCC13-62-derived peptide (MFP-1) on *Escherichia coli* viability. Viable *E. coli* counts after 24h incubation in LB broth containing **(A)** fermented navy bean extract (FBE) or **(B)** PCC13-62-derived peptide (MFP-1) at concentration of 0–400 µg/mL, expressed as log CFU/mL. Statistical significance was determined by one-way ANOVA followed by Dunnett's post hoc test, with comparisons made against the untreated control (0 µg/mL). Asterisks denote statistical significance (* $p < 0.05$, ** $p < 0.01$, *** $p < 0.001$; ns, not significant).
